# Structured Triacylglycerol with Optimal Arachidonic Acid and Docosahexaenoic Acid Content for Infant Formula Development: A Bio-Accessibility Study

**DOI:** 10.3390/foods13172797

**Published:** 2024-09-02

**Authors:** Luis Vázquez, Blanca Pardo de Donlebún, Alejandra Gutiérrez-Guibelalde, Assamae Chabni, Carlos F. Torres

**Affiliations:** 1 Department of Production and Characterization of Novel Foods, Institute of Food Science Research (CIAL, CSIC-UAM), C/Nicolas Cabrera 9, Cantoblanco Campus, Autonomous University of Madrid, 28049 Madrid, Spain; luis.vazquez@uam.es (L.V.); alejandra.gutierrezguibelalde@gmail.com (A.G.-G.); assamae.chabni@uam.es (A.C.); 2 Department of Bioactivity and Food Analysis, Institute of Food Science Research (CIAL, CSIC-UAM), C/Nicolas Cabrera 9, Cantoblanco Campus, Autonomous University of Madrid, 28049 Madrid, Spain; blanca.pardo@csic.es

**Keywords:** acidolysis, arachidonic acid, bio-accessibility, bronchopulmonary dysplasia, docosahexaenoic acid, infant formula, lipase, premature infant

## Abstract

Polyunsaturated fatty acids (PUFAs), especially arachidonic acid (ARA) and docosahexaenoic acid (DHA), are extremely important fatty acids for brain development in the fetus and early childhood. Premature infants face challenges obtaining these two fatty acids from their mothers. It has been reported that supplementation with triacylglycerols (TAGs) with an ARA:DHA (*w*/*w*) ratio of 2:1 may be optimal for preterm infants, as presented in commercial formulas such as Formulaid™. This study explored methods to produce TAGs with a 2:1 ratio (ARA:DHA), particularly at the more bioavailable *sn*-2 position of the glycerol backbone. Blending and enzymatic acidolysis of microalgae oil (rich in DHA) and ARA-rich oil yielded products with the desired ARA:DHA ratio, enhancing *sn*-2 composition compared to Formulaid™ (1.6 for blending and 2.3 for acidolysis versus 0.9 in Formulaid™). Optimal acidolysis conditions were 45 °C, a 1:3 substrate molar ratio, 10% *Candida antarctica* lipase, and 4 h. The process was reproducible, and scalable, and the lipase could be reused. In vitro digestion showed that 75.5% of the final product mixture was bio-accessible, comprising 19.1% monoacylglycerols, ~50% free fatty acids, 14.6% TAGs, and 10.1% diacylglycerols, indicating better bio-accessibility than precursor oils.

## 1. Introduction

The relevance of polyunsaturated fatty acids (PUFAs) in the diet is widely recognized, with ω-6 and ω-3 PUFAs playing crucial roles in preventing syndromes like cardiovascular diseases and neurodegenerative disorders [1]. PUFAs, specifically arachidonic acid (ARA) (ω-6) and docosahexaenoic acid (DHA) (ω-3), are essential for the development of the nervous system during fetal gestation and lactation, making up a substantial portion of brain fatty acids [2]. Brain development in the third trimester of pregnancy and the early neonatal period is rapid and dramatic, suggesting a serious problem for infants born prematurely, especially those who cannot be breastfed [3]. According to the World Health Organization, an estimated 13.4 million babies were born preterm in 2020 (before 37 completed weeks of gestation), and approximately 1 million died from birth complications [4]. Moreover, many of those who survive present disabilities and learning problems related to delayed maturation of the nervous system and other conditions such as retinopathy of prematurity (ROP), bronchopulmonary dysplasia (BPD), or infections. The fortification of breast milk with ARA and DHA in preterm and low-birth-weight infants is crucial [5], as they have increased requirements due to an insufficient accumulation during pregnancy, risking the neurological development of the child [6].

Infant formulas have always included ARA and DHA in their composition, although there has been ongoing debate about their concentration. A clinical study from 2015 concluded that preterm infants fed with an ARA:DHA ratio of 2:1 have better plasma PUFA concentrations and psychomotor development [7]. That year, Bernhard, et al. [8] found higher ARA levels than DHA in the placenta until the 33rd week of pregnancy, linking low ARA:DHA ratios to severe BPD. Heath, Klevebro and Wood [2] indicated that non-breastfed infants fed with a 2:1 ARA:DHA ratio achieved neurodevelopmental outcomes similar to breastfed infants. Likewise, Hellström, et al. [9] concluded that this ratio is highly promising in reducing the risk of severe ROP compared to a 1:1 ratio and the control group. Specialized products such as Formulaid™, derived from fungi and microalgae, apply ARA:DHA ratios of 1:1 and 2:1 for use in infant formula. Formulaid™ was utilized in the trial by Hellström, Nilsson, Wackernagel, Pivodic, Vanpee, Sjöbom, Hellgren, Hallberg, Domellöf and Klevebro [9] and is currently employed as an intervention product in a clinical trial at Oslo University Hospital concluding in 2029. This trial evaluates brain maturation and growth quality in premature infants with essential fatty acid deficiency.

Despite having an appropriate ARA:DHA ratio, the bio-accessibility of this product in preterm infants is uncertain due to the immaturity of their digestive system and the low activity of pancreatic lipase enzyme, showing reduced absorption of ARA and DHA [10]. In this regard, different strategies have been studied to enhance the absorption of these fatty acids, such as parenteral nutrition, the use of phospholipids, pre-digested triacylglycerols (TAGs) [11], or the synthesis of structured lipids. The design of structured lipids allows the concentration of specific fatty acids in a single compound, which enhances their nutritional and physicochemical properties through a specific positional distribution that makes their absorption and metabolism more efficient [12]. Essential fatty acids are absorbed most efficiently when located at the *sn*-2 position of the TAGs. During digestion, pancreatic lipase hydrolyzes the *sn*-1 and *sn*-3 positions of the TAGs, releasing fatty acids and 2-monoacylglycerols (2-MAGs), which are easily absorbed because they form mixed micelles with bile acids and are better preserved than free fatty acids (FFAs), which can form insoluble soaps [13]. Indeed, in breast milk, DHA and ARA fatty acids are mostly located at the *sn*-2 position of TAGs (60% and 45, respectively); however, in infant formulas, they are typically found at lower levels (34% and 28%) [14]. Additionally, when ARA and DHA are located at *sn*-1,3 positions, they induce resistance to pancreatic lipase, thus resulting in a low absorption of these fatty acids in these positions [15].

The production of structured lipids involves different types of reactions, such as alcoholysis, acidolysis, or interesterification, which can be carried out through chemical or enzymatic catalysis. The enzymatic pathway using lipases can be favorable due to the mild pressure and temperature conditions it entails, as well as the specificity that some lipases exhibit in discriminating between different fatty acids or specific positions within the TAG molecule [16]. Some studies have explored enzymatic interesterification for the production of human milk fat substitutes (HMFSs) for use in infant formulas. With this aim, Turan, et al. [17] synthesized a structured lipid rich in ARA and DHA from the commercial oils *ARASCO* (rich in ARA) and *DHASCO* (rich in DHA) with an *ARASCO:DHASCO* ratio of 3:2 at 60 °C for 3 h of reaction time using the *Rhizomucor miehei* (RM) enzyme as a catalyst. On the other hand, enzymatic acidolysis is one of the most developed strategies for producing structured lipids concentrated in specific PUFAs, including the incorporation of ARA and DHA for HMFS synthesis. In various studies, optimal temperatures range from 50 to 65 °C, with a molar ratio of substrate oil:FFAs around 1:3, and typically employing RM, which exhibits specificity for the *sn*-1 and *sn*-3 positions [18,19]. Other studies have shown very similar results using *Candida antarctica* lipase (CA) as a catalyst [20].

An effective method to evaluate the bio-accessibility of lipid formulations is through in vitro digestion [21]. These in vitro models allow for closely simulating gastrointestinal conditions while being simple, economical, fast, and reproducible. They also enable the monitoring of various parameters and sample collection during the process to ensure accurate follow-up, avoid inter-individual variation, and overcome ethical or experimental limitations [21,22,23,24,25]. These advantages make them a good alternative to in vivo models in animals or clinical trials in humans. Although in vivo models provide more physiological conditions, they have disadvantages such as high economic cost, long time requirements, influence of external factors, low reproducibility, difficulty in monitoring, and ethical issues [21,23,25].

The main objective of this study was the production of structured TAGs with a high concentration of ARA and DHA, along with an ARA:DHA ratio (*w*/*w*) of at least 2:1, aiming for the closest approximation to this value. In addition, the 2:1 ARA:DHA ratio (*w*/*w*) at the *sn*-2 position of the TAGs was also maintained. Finally, the bio-accessibility of the structured lipid produced was also evaluated in an in vitro digestion model. This product is intended to be used in infant formula for premature infants.

## 2. Materials and Methods

### 2.1. Materials

The microalgal oil from *Schizochytrium* sp., with a high content of DHA, was obtained from Progress Biotech (Capelle aan den Ijssel, The Netherlands). The oil with high content of ARA was obtained from Penta Manufacturing Company (Caldwell, NJ, USA). Both oils were stored under nitrogen atmosphere at −20 °C to minimize potential oxidation. For clarity, the microalgal oil and the ARA-rich are designated as DHA-oil and ARA-oil, respectively, hereafter in this study. The enzymes *Eversa^®^ Transform 2.0* from *Aspergillus oryzae*, Lipozyme RM IM from *Rhizomucor miehei* (RM), Lipozyme TL IM from *Thermomyces lanuginosus* (TL), and Novozym 435 from *Candida antarctica* (CA) were donated by Novozymes A/S (Bagsvaerd, Denmark). The reference ARA:DHA 2:1 formula, Formulaid™, was supplied by DSM (Heerlen, Limburg, Belgium).

Reagents used for in vitro digestion: Trizma^®^, pepsin, maleic acid, bile salts and cholesterol were purchased from Sigma-Aldrich (St. Louis, MO, USA). Phosphatidylcholine from egg yolk was supplied by Lipoid (Ludwigshafen, Germany), food-grade phospholipase A2 from *Streptomyces violaceoruber* (103 U mg^−1^) was supplied by Nagase Chemtex Corporation, Fukuchiyama Factory (Kyoto, Japan). Pancreatin from porcine pancreas was purchased from MP Biomedicals, LLC (Irvine, CA, USA). Hydrochloric acid, sodium chloride, and calcium chloride were from Panreac (Barcelona, Spain). Rabbit gastric lipase was supplied by Lipolytech (Marseille, France) and stored in freezer storage.

KOH (ultrapure), phenolphthalein (1% solution in ethanol), and diethyl ether were purchased from Scharlau (Sentmenat, Spain). NaOH (pure), potassium hydrogen phthalate (ultrapure), methanol (≥99.5%), and absolute ethanol were obtained from Panreac (Castellar del Vallés, Spain). BF_3_-methanol and BSTFA used for derivatization were supplied by Supelco (Bellefonte, PA, USA), and methyl tert-butyl ether (MTBE), chloroform, petroleum ether, and hexane-95% were obtained from Macron (Deventer, The Netherlands), and formic acid (98%) from Panreac (Barcelona, Spain). All these solvents were of High performance liquid chromatography (HPLC) and gas chromatography (GC)grade. Silica SPE columns (3 mL, 500 mg) were purchased from Sigma-Aldrich (St. Louis, MO, USA).

### 2.2. Methods

#### 2.2.1. Analysis of Fatty Acid Profile

The fatty acid profile of initial oils, its blending, and Formulaid™ was analyzed by gas chromatography (GC), according to the method of Vázquez de Frutos, et al. [26]. In a previous step, fatty acids were transformed into their corresponding fatty acid methyl esters (FAMEs). The methylation of fatty acids was carried out according to the AOAC Official Method 996.01 [27], using NaOH/methanol solution (0.5 M) and BF_3_/methanol solution (~14%, *w*/*v*) as catalysts. The identification and quantification of FAMEs were carried out in an Agilent 6850 Network GC System (Agilent, Avondale, AZ, USA), coupled to an FID detector and Agilent 6850 autosampler. The capillary column was an HP-88 (30 m, 0.25 mm i.d.) (Agilent, Avondale, AZ, USA). An injection volume of 1 μL and a 20:1 split ratio was used. The injector and detector temperatures were 220 and 250 °C, respectively. The temperature program started at 50 °C, rising to 220 °C at 15 °C·min^−1^. The final temperature (220 °C) was held for 10 min. The identification of FAMEs was based on the retention times and the relative area percentages of No. 3 PUFA reference standard (47085-U), obtained from Supelco (Bellefonte, PA, USA).

#### 2.2.2. Analysis of Positional Distribution of Fatty Acids

The positional distribution of fatty acids in the TAG molecule was performed according to the method by Watanabe, et al. [28], with modifications. A 200 mg measure of oil, 2 g of absolute ethanol and 0.088 g of CA lipase were mixed. The mixture was kept at 30 °C for 3 h in an orbital shaker at 150 rpm. After the ethanolysis reaction, the mixture was filtered with a 5 mL syringe and a 0.45 µm nylon filter to separate the enzyme from the reaction medium. Ethanol was evaporated under a stream of nitrogen until constant weight.

The separation of fractions, i.e., fatty acid ethyl esters (FAEEs), diacylglycerols (DAGs), and 2-MAGs was performed by solid-phase extraction (SPE), employing 500 mg silica cartridges (Sigma-Aldrich, St. Louis, MO, USA). The cartridge was pre-equilibrated with 10 mL of hexane:ethyl acetate in a ratio of 8:2 (*v*/*v*). Then, another 10 mL of this solvent was added to elute the FAEEs. Subsequently, another 20 mL of this solvent was added to elute the 1,2-DAG fraction. Finally, 10 mL of pure ethyl acetate was added to elute the 2-MAG fraction, which was then evaporated under a nitrogen stream until constant weight. The 2-MAGs were methylated and analyzed by GC following the methodology described in Section 2.2.1. The fatty acid profile resulting from the 2-MAG fraction corresponds to the *sn*-2 position of the TAGs.

The fatty acid profile of the *sn*-1 and *sn*-3 positions of the TAGs was determined according to the formula

(1) $$sn˗1,\;3=\;\frac{\left[(3\times \mathrm{total}\;\mathrm{fatty}\;\mathrm{acids})-(sn˗2)\right]}{2}$$

#### 2.2.3. Lipase-Catalyzed Hydrolysis of DHA-Oil

Enzymatic hydrolysis of DHA-oil was carried out by using *Eversa^®^ Transform 2.0* lipase as a catalyst. Reactions were performed using an IKA RCT Basic temperature control system (Staufen, Germany) with mechanical agitation by a magnetic stirrer. According to preliminary studies of the group, 50 g of oil were mixed with 50 g of distilled water in a 250 mL flask at 45 °C with the mixture kept under vigorous and constant agitation (~300 rpm) to ensure that the two immiscible phases (oil and water) were always in contact. Then, 2.5% (*w*/*w*) of *Eversa^®^ Transform 2.0* lipase relative to the weight of oil was added to the mixture, and after 32 h, an additional 2.5% (*w*/*w*) of lipase was added, continuing the reaction until 96 h [11].

To recover the product, the mixture was centrifuged at 40 °C and 11,000 rpm for 5 min using a Heraeus Multifuge 3SR+ centrifuge (Thermo Fisher Scientific, Waltham, MA, USA). Hence, the aqueous phase was removed, and the oily phase was washed with 50% (*w*/*w*) distilled water and centrifuged again under the same conditions. The oily phase was then evaporated to a VWR RV 10 control rotary evaporator (IKA, Staufen, Germany) set at 50 °C and <1 mbar to completely remove residual water. The product, consisting of ~96 wt% FFAs, was stored in absence of light and under a nitrogen atmosphere to minimize potential oxidation. The DHA-rich free fatty acid product was used as raw material for subsequent acidolysis reactions.

#### 2.2.4. Lipase-Catalyzed Acidolysis

Lipase-catalyzed acidolysis reactions were carried out using ARA-oil (TAGs) and FFAs from the hydrolysis of DHA-oil as substrates. Various variables such as temperature, molar ratio of substrates, and type of lipase were investigated. Lipases from CA, TL, and RM were employed as catalysts. Molar ratios of 1:1, 1:3, and 1:6 for the substrates ARA-oil:DHA-FFAs were investigated using RM lipase. A 10 g measure of the reaction mixture was placed in a 60 mL vial at 45 and 55 °C, with magnetic stirring at 380 rpm and 10% (*w*/*w*) of lipase. Aliquots of 350 µL were taken at reaction times of 1, 2, 4, 8, 24, and 48 h, which were dissolved in 2 mL of hexane. The samples were filtered using a 5 mL syringe and a 0.45 µm nylon filter to separate the enzyme and then evaporated under a nitrogen stream for subsequent analysis.

#### 2.2.5. Analysis of Total FFAs

The acidity, which corresponds to the FFAs content of the hydrolysis and acidolysis products, was analyzed by volumetric titration based on ISO 660:2020 [29] with modifications. Samples of 50–100 mg obtained at different time intervals of reactions were dissolved in a 25 mL diethyl ether–ethanol solution (1:1, *v*/*v*) and then titrated with KOH at concentrations ranging from 0.01 to 0.05 M. Phenolphthalein (1%, *w*/*v*) in ethanol was used as the indicator.

#### 2.2.6. Neutralization of Acidolysis Aliquots and Products

A chemical neutralization of the aliquots and products obtained through enzymatic acidolysis was conducted with the aim of removing the FFAs present in the samples and thus purifying the synthesized structured TAGs prior to analysis by GC according to Section 2.2.1. The procedure was fine-tuned using the methodology described by Araújo, et al. [30]. The sample was mixed in a 15 mL *Falcon* tube with 5 mL of hexane and with the volume corresponding to the KOH used to determine its acidity according to Section 2.2.5. The mixture was gently shaken, and then another 5 mL of hexane were added, and it was gently shaken again. Subsequently, it was centrifuged at 4500 rpm at 25 °C for 5 min using a Heraeus Multifuge 3SR+ centrifuge (Thermo Fisher Scientific, USA). Finally, the hexane phase containing the fraction of purified TAGs, i.e., without FFAs, was collected and evaporated under a nitrogen stream until constant weight.

#### 2.2.7. In Vitro Gastrointestinal Digestion Model

The in vitro gastrointestinal digestion model was carried out according to the procedure by Chabni, Bañares, Reglero and Torres [23]. This methodology comprises two stages: (i) gastric and (ii) intestinal digestion [11].

##### Gastric Digestion

Structured TAGs were prepared for gastric digestion. A 1.86 g sample was mixed with 7.44 mL of gastric phase simulation fluid in a digester set to 37 °C and stirred at 900 rpm for 10 min using a mechanical stirrer. Prior to this, the mixture was sonicated with an ultrasound probe for 1 min at 70% power. Gastric digestion was initiated by adding 1.16 mL of an enzyme solution containing 400 mg of rabbit gastric lipase and 24 mg of porcine pepsin dissolved in 2.5 mL of distilled water. The digestion was performed at 37 °C with continuous stirring at 900 rpm for 60 min.

To monitor lipid hydrolisis, 400 μL samples were withdrawn at 10, 20, 30, 40, and 60 min. At 60 min, a sample of 2.925 mL was also collected to ensure 1 g of fat was available for intestinal digestion.

##### Intestinal Digestion

After gastric digestion, 26 mL of Trizma-maleate buffer (0.1 M, pH 7.5) was added to the stirred mixture. To simulate biliary secretion, a solution was prepared consisting of 500 mg of bile salts, 200 mg of egg yolk phosphatidylcholine, 40 mg of cholesterol, 1 mL of a 325 mM CaCl_2_ solution, 3 mL of a 3.25 mM NaCl solution, and 20 mL of 0.1 M Trizma-maleate buffer, pH 7.5. This mixture was homogenized with ultra-turrax T18 basic IKA (Staufen, Germany) for 2 min at 3500 rpm. Afterwards, the simulation bile secretion was added to the resulting emulsion after gastric digestion, and all of it was homogenized again with ultra-turrax for 2 more min at 3500 rpm. Intestinal digestion began by adding a solution of fresh pig pancreatin extract prepared from 100 mg of pancreatin in 4 mL of Trizma-maleate buffer 0.1 M pH 7.5, stirred at 900 rpm for 10 min and centrifuged at 1600 G, at 5 °C for 15 min. The supernatant was added to the reaction medium together with 2.5 mL of food-grade phospholipase A2. The intestinal phase was carried out for 60 min at 37 °C and 900 rpm.

To examine the changes in lipid products during the hydrolysis process in intestinal digestion, 1 mL aliquots were collected at 0, 2, 5, 10, 20, 30, 45, and 60 min, with the final sample representing the end of digestion. The 0 min aliquot was taken before the addition of the pancreatin solution.

##### Phase Separation after In Vitro Digestion

After 60 min of intestinal digestion, the products were centrifuged at 4500 rpm and 37 °C for 45 min according to the method described by Martin, et al. [31]. After centrifugation, three phases were obtained: an upper oily phase (OP), formed by the undigested lipid fraction; an intermediate aqueous fraction, called the micellar phase (MP), which contains the lipid fraction digested in the form of micellar and vesicular structures; and a precipitate phase (PP), which consists of a lower phase containing insoluble compounds at 37 °C. These three phases were separated from each other for the subsequent extraction of the lipids present in each of them.

##### Lipid Extraction

The total lipids contained in the three phases (OP, MP, and PP) were extracted from the samples in three sequential steps using solvent mixtures with increasing polarity in a solvent:sample ratio of 3:1 (*v*/*v*) and centrifuging for 10 min at 14,500 rpm each time, with the exception of the PP, which was extracted with a solvent:sample ratio of 6:1 (*v*/*v*), according to the methodology byCorzo-Martínez, et al. [32]. The solvents used were n-hexane/methyl-tert-butyl-ether (MTBE) (50:50, *v*:*v*); MTBE/petroleum ether (50:50, *v*:*v*); and petroleum ether/ethanol (1:0.6, *v*:*v*). After each extraction, the supernatant was collected in an empty vial, and possible impurities were allowed to decant. Later, the supernatant was transferred to another previously weighed vial and evaporated under a stream of nitrogen to a constant weight of the residue (w.r.). Finally, the samples were diluted with acidified chloroform (0.1% formic acid) to a final concentration of 6 mg mL^−1^ before injection in the GC system.

#### 2.2.8. Analysis of Lipids from Gastrointestinal Digestion

Lipid samples obtained from gastrointestinal digestion were analyzed by gas chromatography (GC). GC consisted of an Agilent 7820A gas chromatograph (Santa Clara, CA, USA) with on-column injection coupled to flame ionization detector (FID). The chromatographic separation was based on the method developed by Torres, et al. [33]. An HP-5MS capillary column (7 m × 0.25 mm internal diameter × 0.25 μm) was used. The injection volume was 0.2 μL. Temperatures of injector and detector were 50 and 340 °C, respectively. The program of temperatures started at 60 °C, increasing at 42 °C min^−1^ until 250 °C. This temperature was maintained for 20 min and then increased up to 340 °C at 25 °C min^−1^, which was maintained for 20 min. The flow rate of carrier gas (helium) was maintained at 1 mL/min.

The data are expressed as a percentage of TAG, DAG, MAG, and FFA with respect to the weight of the residue (w.r.) obtained after lipid extraction, according to the following equation:

(2) WcompWw.r.×100

where W_comp_ is the weight of each type of lipid and W_w.r._ is the weight of the residue obtained after lipid extraction.

The distribution of each lipid class among three phases was determined by combining the relative weight of each phase and composition according to the following equation:

*%* of *A* = weight percent of oil phase × (percentage of *A* in oil phase/100) + weight percentage of the micellar phase × (percentage of *A* in the micellar phase/100) + weight percentage of the precipitated phase × (percentage of *A* in the precipitated phase/100) (3)

where *A* refers to any of the types of lipids analyzed in this study.

## 3. Results and Discussion

### 3.1. Fatty Acid Profile and Positional Distribution of Initial Oils, Their Blending, and Formulaid™

The first strategy carried out to obtain an oil with an ARA:DHA (*w*/*w*) ratio of 2:1 was to blend ARA-oil and DHA-oil in a 2:1 proportion, since, as shown in Table 1, the % of ARA and DHA were ~54% in ARA-oil and DHA-oil, respectively. Table 1 shows the fatty acid profile and positional distribution of the starting oils, the blending, and the commercial product Formulaid™.

ARA:DHA (*w*/*w*) ratio of 2:1 has been prioritized as an objective, not only in total TAGs, but also in the *sn*-2 position of the TAGs mixture. Preliminary studies have shown improved absorption of fatty acids located at this position relative to those located at *sn*-1,3. In addition, when ARA and DHA are at *sn*-1,3 they induce resistance to pancreatic lipase, and therefore, low absorption can be expected under these conditions [15].

As observed in Table 1, the blending process is a very rapid and simple procedure to achieve a product with an ARA:DHA (*w*/*w*) ratio in total TAGs close to the target (1.9). However, this ratio decreased to 1.6 at the *sn*-2 position. Although the commercial product Formulaid™ contains an ARA:DHA (*w*/*w*) ratio close to 2, matching with the aim of this study, its positional distribution were not described in the literature. Its precursors, *ARASCO* and *DHASCO*, contain 30 and 44–50% of ARA and DHA present in the *sn*-2 position, respectively [34]. Furthermore, a blending in a 2:1 ratio of ARASCO and DHASCO produces a fatty acid profile similar to that found Formulaid™. This confirmed that the ratio at *sn*-2 position of the blended product utilized in this study is more favorable than that observed in Formulaid™, being 1.6 and 0.9, respectively.

### 3.2. Acidolysis

**Figure 1:**
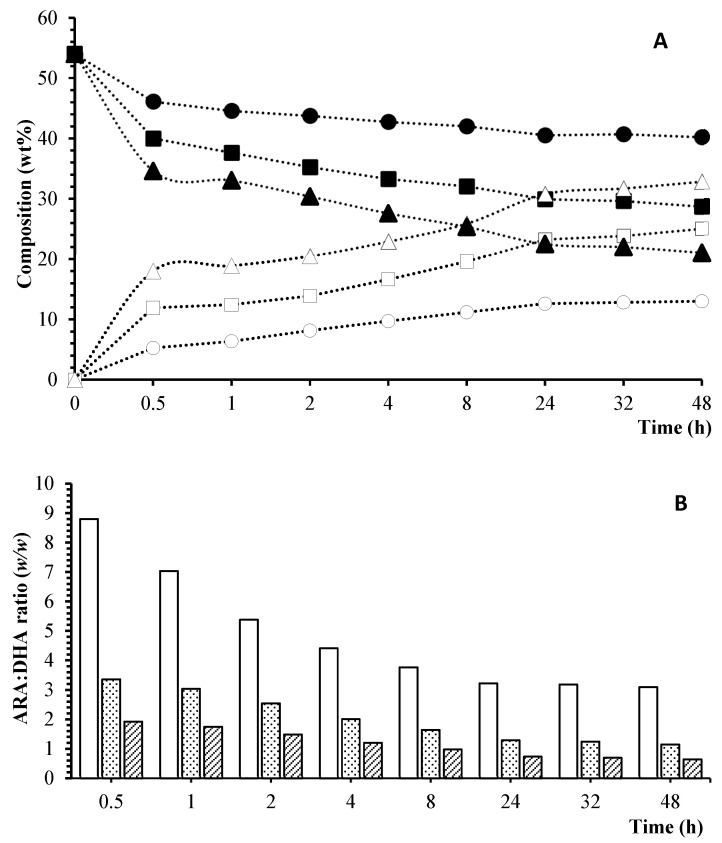
Acidolysis at 55 °C with RM lipase at different ARA-oil:DHA-FFA ubstrate molar ratios. (**A**) Composition (wt%) of ARA and DHA. (●) 1:1—ARA; (■) 1:3—ARA; (▲) 1:6—ARA; (○) 1:1—DHA; (□) 1:3—DHA; (Δ) 1:6—DHA. (**B**) ARA:DHA (*w*/*w*) ratio. (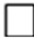) 1:1; (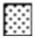) 1:3; (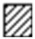) 1:6.

Acidolysis reactions were carried out using TAGs from ARA oil and FFAs from DHA oil as substrates. Firstly, the effect of the ARA-oil:DHA-FFAs molar ratio of substrates was investigated using RM lipase as catalyst. This enzyme was selected because it was widely employed in acidolysis processes aimed at synthesizing structured lipids for infant formulae [19,30,35,36]. RM lipase exhibits *sn*-1,3 positional specificity, and thus, in theory, it can more effectively preserve a high concentration of ARA at *sn*-2, facilitating the achievement of the set objective. Figure 1 depicts the wt% of ARA and DHA, along with the ARA:DHA (*w*/*w*) ratio in the purified TAGs, across different reaction times, considering the various substrate molar ratios investigated (1:1, 1:3 and 1:6).

As shown in Figure 1A, the incorporation of DHA into TAGs increased progressively over time, resulting in a gradual decrease in ARA. This variation of DHA and ARA became more pronounced as the ARA-oil:DHA-FFAs molar ratio increased. In agreement with other authors, increasing the molar ratio of FFAs/TAGs promotes the equilibrium of the reaction and results in the effective incorporation of the desired fatty acids into the oils [35,37]. Thus, the 1:1 molar ratio resulted in low incorporation of DHA into TAGs (13% at 48 h), while with the 1:3 molar ratio, the % of DHA was similar to that of ARA at 48 h (25–29%), and with the 1:6 ratio, the concentrations of DHA and ARA were practically equal (~25%) at 8 h.

Figure 1B shows that the 1:1 molar substrate ratio did not result in sufficient incorporation of DHA to achieve ARA:DHA (*w*/*w*) ratios close to 2. Although a decrease in this ratio was observed over time, it remained consistently above 3. On the contrary, when a large excess of free DHA was used in the reaction, i.e., a 1:6 molar ratio, the optimal ARA:DHA (*w*/*w*) ratio of 2:1 was reached at 30 min of reaction, decreasing thereafter. From the perspective of avoiding excessive consumption of free DHA as a substrate, the 1:6 molar ratio was not considered adequate. Nevertheless, the 1:3 substrate molar ratio was selected as optimal, as it resulted in an ARA:DHA (*w*/*w*) ratio of 2:1 within 4 h.

**Figure 2:**
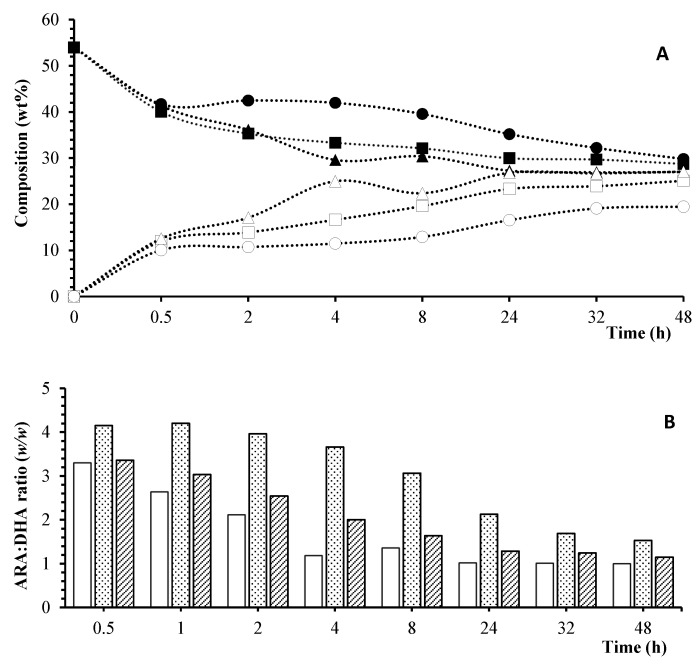
Acidolysis at 55 °C and 1:3 ARA-oil:DHA-FFAs substrate molar ratio with different lipases. (**A**) Composition (wt%) of ARA and DHA. (●) TL—ARA; (■) RM—ARA; (▲) CA—ARA; (○) TL—DHA; (□) RM—DHA; (Δ) CA—DHA. (**B**) ARA:DHA (*w*/*w*) ratio. (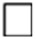) CA; (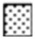) TL; (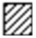) RM.

Subsequently, other lipases with different positional specificity, such as TL and CA lipases, were investigated at 55 °C, with an ARA-oil:DHA-FFAs molar ratio of 1:3. These lipases were selected because they were used in previous lipase-catalyzed acidolysis studies [35]. Theoretically, TLs exhibit positional specificity for *sn*-1,3 positions of TAGs, similarly to RM. On the other hand, although CA may present a preference for *sn*-1,3 positions [38], its positional specificity is lower than TL and RM, being capable of catalyzing reactions, including acidolysis, at the three positions of TAGs. Figure 2 depicts the wt% of ARA and DHA, along with the ARA:DHA (*w*/*w*) ratio in the purified TAGs, across different reaction times, considering the various lipases investigated.

In a subsequent experiment, a temperature reduction to 45 °C was attempted for CA and RM, maintaining a molar ratio of ARA-oil:DHA-FFAs of 1:3. As was expected, the reaction rate decreased at 45 °C compared to 55 °C. High temperatures promote the conversion of substrates in enzyme-catalyzed reactions by enhancing the solubility of the substrates, decreasing the viscosity of the reaction medium and favoring mass transfer. However, an increase in temperature also leads to thermal deactivation of the enzyme [39]. Thus, at 45 °C, the reaction time required to achieve the target ARA:DHA (*w*/*w*) ratio of 2:1 increased to 4 h for CA and 8 h for RM. However, the temperature of 45 °C was selected as optimal with the aim of using milder conditions and reducing energy consumption. The positional distribution of structured TAGs produced at optimal conditions was determined (see Table 2).

As shown in Table 2, RM provided an ARA:DHA (*w*/*w*) ratio of 3.8 at 45 °C, which deviated from the proposed target of ~2. This fact can be explained by the *sn*-1,3 regiospecificity of RM, which resulted in a low incorporation of DHA at *sn*-2 of TAGs (~11%). However, CA lipase, with lower positional specificity than RM, was able to incorporate approximately 17% of DHA at *sn*-2, resulting in an ARA:DHA (*w*/*w*) ratio of 2.3, which is close to the target. For this reason, CA lipase was selected as the optimal catalyst for the reaction, being also the fastest and resulting in a more favorable ARA:DHA (*w*/*w*) ratio at *sn*-2 than that obtained with the blending product (1.6) and that of the commercial formula Formulaid™ (0.9).

### 3.3. Scale-Up

Three scale-up processes were successively carried out under identical conditions, using 50 g of ARA-oil:DHA-FFAs mixture with a molar ratio of 1:3, at 45 °C, and employing CA lipase as catalyst for 4 h. Moreover, lipase was pressure-filtered under a stream of nitrogen and reutilized between processes. It should be noted that the processes were highly reproducible. Hence, although small differences were observed in the initial reaction times, by 4 h, the concentrations of both ARA (35–36%) and DHA (18–19%) were practically identical, with the ARA:DHA (*w*/*w*) ratio remaining stable around 2. This also indicates that CA lipase maintains its enzymatic activity after the three scale-up processes, which is an advantage from a possible industrial scaling perspective.

Table 3 shows the positional distribution of the structured TAGs obtained in the scale-up processes. High reproducibility was observed in the composition at *sn*-1,3 and *sn*-2 positions among the three studied processes. Additionally, a slight increase in the ARA:DHA (*w*/*w*) ratio was observed in the scale-up (~2.3) compared to the lab-scale process (~1.9). This ratio at the *sn*-2 position was ~2.4, very similar to that of the total TAGs and also to that obtained in the laboratory-scale process (~2.4).

**Figure 3:**
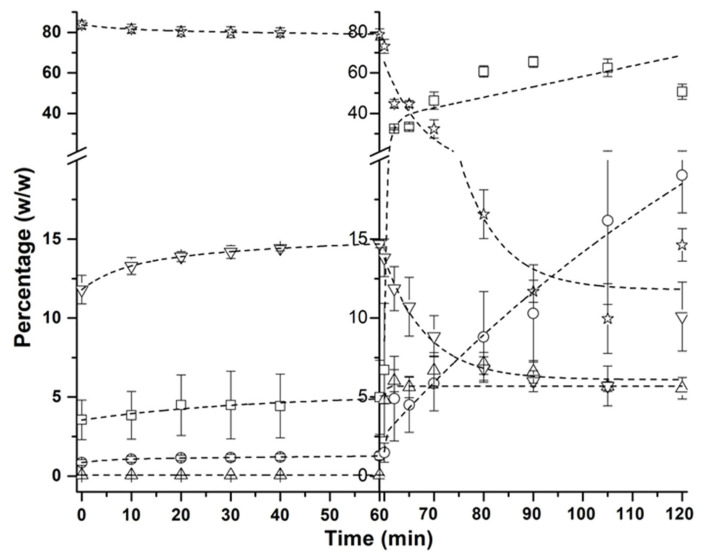
Time course of in vitro gastric and intestinal digestion of the scale-up product. 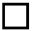 FFA, 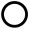 MAG, 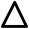 cholesterol, 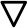 DAG, 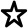 TAG.

Furthermore, the neutralization step conducted after the three scale-up processes was highly effective, as the residual content of FFAs were less than 5% (Figure 3, time 0). Thus, 56.3 g of final product was obtained, comprising mainly TAGs with an ARA:DHA (*w*/*w*) ratio of 2.3–2.4, including the *sn*-2 position.

### 3.4. In Vitro Gastrointestinal Digestion of the Scale-Up Product

Figure 3 depicts the dynamic changes in the composition of the scale-up product during the digestion process, highlighting the significant transformation of TAGs into more bio-accessible forms such as FFAs and MAGs. In the initial gastric digestion stage, the percentages of FFAs, MAGs, and DAGs slightly increased.

A slight decrease in the TAGs percentage was also observed. However, the most relevant variations in lipid composition occurred during the subsequent intestinal digestion stage. It should be noted that gastric lipase retained activity in the intestinal stage due to its operational pH range of 2–8, which also contributed to the hydrolysis of TAGs [11].

During the intestinal phase, TAGs content decreased by up to 14.6%, alongside a reduction in DAGs to 10.1%. Consequently, MAGs and FFAs increased to 19.1% and 50.7%, respectively. In this regard, these two variables indicate adequate bio-accessibility [40], although relatively high levels of undigested DAGs and TAGs were also attained. It is worth mentioning that there is a significant improvement in digestibility compared to precursor oils, the digestion of which was carried out in a previous study and in which it has been found that the latter do not reach 10% MAGs, while in the structured lipid, it is close to 20%.

**Figure 4:**
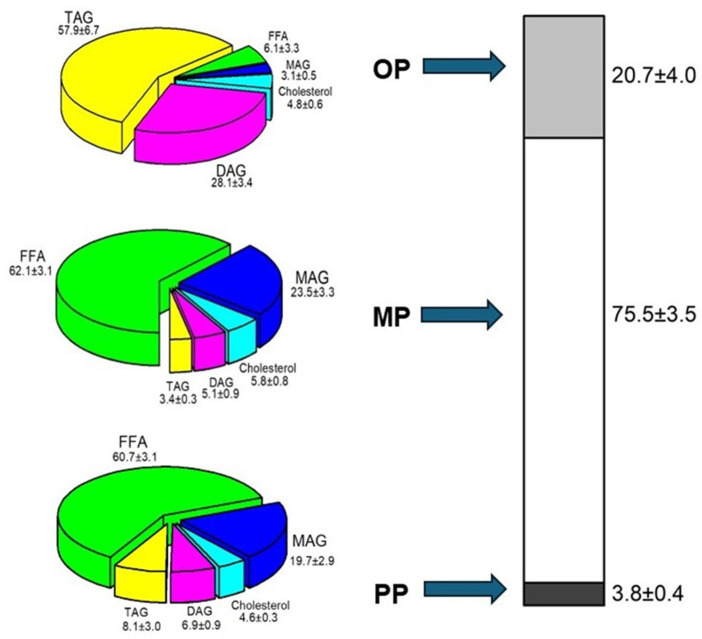
Phase distribution and composition (wt%) after digestion of the scale-up product. 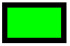 FFA: free fatty acid, 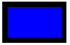 MAG: monoacylglycerol, 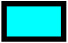 Cholesterol, 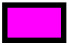 DAG: diacylglycerol, 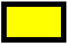 TAG: triacylglycerol. 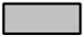 OP: oil phase, 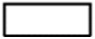 MP: micellar phase, 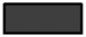 PP: precipitate phase.

Moreover, Figure 4 shows the composition of the oily phase (OP), micellar phase (MP), and precipitate phase (PP) after centrifugation. It should be indicated that 75.5% of the digestion product was found in the MP, composed mainly of MAGs (62.1%) and FFAs (23.5%), which form part of the micelles, thereby constituting the bio-accessible fraction. On the other hand, 20.7% of the OP was obtained, composed mainly of TAGs (57.9%) and DAGs (28.1%) that had not been digested, thus representing the non-bio-accessible fraction. Only 3.5% of the PP was obtained, formed by FFAs (60.7%) and MAGs (19.7%) that were insoluble at 37 °C. From these data, it was observed that most of the MAGs and FFAs were found in the MP, while the remaining DAGs and TAGs mainly appeared in the OP. In contrast, after gastrointestinal digestion in a previous study, ARA-oil and DHA-oil resulted in an OP of 26.7% and 20.0%, an MP of 69.5% and 70.2%, and a PP of 3.8% and 9.8%, respectively. Therefore, it can also be concluded that the structured TAGs exhibited better bio-accessibility in comparison to the precursor oils.

**Figure 5:**
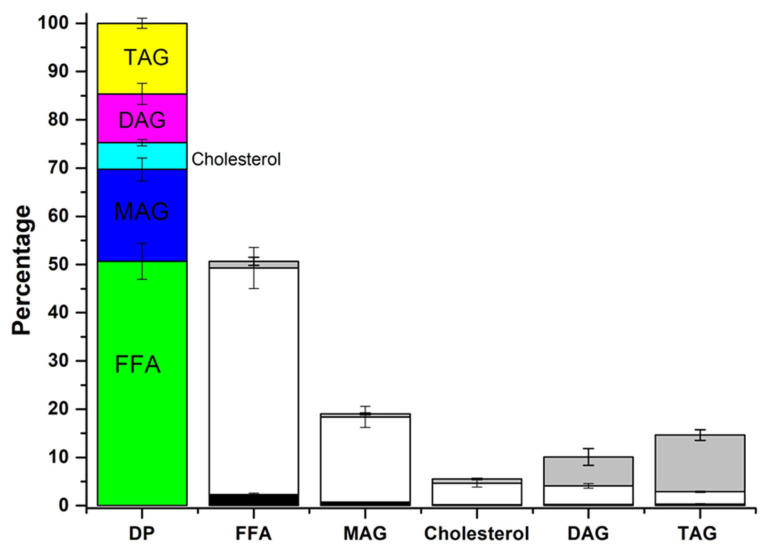
The distribution of each lipid compound among the different phases. 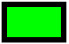 FFA: free fatty acid, 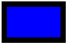 MAG: monoacylglycerol, 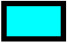 Cholesterol, 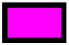 DAG: diacylglycerol, 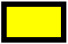 TAG: triacylglycerol. 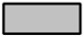 OP: oil phase, 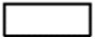 MP: micellar phase, 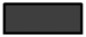 PP: precipitate phase.

The composition of each phase, determined by GC, was used to analyze the distribution of each lipid class between the OP, MP, and PP. However, when examining the percentage of each lipid class in each phase after centrifugation (as shown in the sector diagrams in Figure 4), it is not possible to determine the relative amount or content of each lipid in each phase. Understanding the distribution of each lipid class in each phase is crucial for determining which lipid compounds are more bio-accessible (Figure 5). Among the 50.7% of FFAs present in the DP, the largest fraction is found in the MP (46.6%), and the remaining fraction is distributed between the OP and PP (1.8 and 2.3%, respectively). Likewise, for the MAGs, 17.7% of the 19.1% present in the DP are located in MP. These lipid digestion products, together with cholesterol, also largely present in MP, are solubilized in mixed micelles to make them more bio-accessible [11,41]. Approximately 40% of the total DAGs are located in the MP, suggesting that the MP has excellent emulsifying properties and can incorporate a substantial portion of DAGs.

## 4. Conclusions

This study demonstrated that the blending of ARA-rich oil and microalgae oil can be a fast and simple procedure to obtain an oil with an ARA:DHA (*w*/*w*) ratio of ~2, with a lower ratio of these two fatty acid residues at *sn*-2 position (~1.6). Alternatively, the acidolysis reaction was effective in producing structured TAGs with an ARA:DHA (*w*/*w*) ratio of 1.9–2.3 in the total TAGs and 2.3–2.4 at the *sn*-2 position. This process was highly reproducible and scalable and permitted the reutilization of the lipase. These products significantly improved the ARA:DHA (*w*/*w*) ratio at the *sn*-2 position compared to the commercial formula Formulaid™. In vitro digestion revealed slightly better digestibility that that observed with the precursor oils, with 75% of the digestion product recovered in MP. Although further investigations are needed to probe the bioavailability of ARA and DHA, this new structured lipid provides in a very interesting strategy to incorporate ARA and DHA in a single molecule with a ratio of 2:1 even at the *sn*-2 position, and it could provide an interesting vehicle for these two fatty acids intended to be used in infant formula.

## Figures and Tables

**Table 1:** Fatty acid composition (wt%) and positional distribution of ARA-oil, DHA-oil, blending and Formulaid™ (Trademark). Minor fatty acids contributing to the total fatty acid composition were capric acid, lauric acid, and myristoleic acid.

Fatty Acid (wt%)	ARA-Oil			DHA-Oil			Blending			Formulaid™		
	Total	*sn*-2	*sn*-1,3	Total	*sn*-2	*sn*-1,3	Total	*sn*-2	*sn*-1,3	Total	*sn*-2	*sn*-1,3
Myristic	0.3	0.3	0.3	0.8	0.5	1.0	0.5	0.1	0.0	4.6	0.9	6.5
Palmitic	6.9	1.2	9.8	18.4	5.0	25.1	10.6	3.4	14.2	10.1	1.4	14.5
Palmitoleic	0.3	0.4	0.2	0.5	0.3	0.7	0.4	0.4	0.4	1.0	0.7	1.2
Margaric	0.5	0.1	0.7	0.2	0.1	0.2	0.4	0.4	0.5	0.2	0.2	0.3
Stearic	10.8	1.5	15.5	1.5	0.6	1.9	7.7	1.7	10.7	5.7	0.6	8.2
Oleic	8.6	15.2	5.2	6.9	7.3	6.7	7.7	19.4	1.9	20.5	29.8	15.9
Linoleic	6.1	14.8	1.8	0.9	1.2	0.8	4.4	10.2	1.5	5.3	11.1	2.4
γ-Linolenic	2.8	5.2	1.5	0.3	0.2	0.4	2.8	3.5	2.5	2.5	2.8	2.4
α-Linolenic	1.9	1.1	2.3	0.2	0.2	0.2	0.4	0.7	0.3	0.3	0.2	0.3
Eicosenoic	0.7	0.1	1.0	0.3	0.1	0.6	0.5	0.4	0.5	0.0	0.0	0.0
Behenic	4.6	5.2	4.2	1.2	0.5	1.6	3.3	2.8	3.6	3.2	3.5	3
ARA	54.0	54.0	54.0	0.1	0.6	0.0	35.4	30.9	37.6	27.3	22.5	29.7
Erucic	0.0	0.0	0.0	0.8	0.6	1.0	0.7	0.3	0.9	0.0	0.0	0.0
Eicosapentaenoic	0.0	0.0	0.0	0.7	0.4	0.9	0.4	0.2	0.6	0.7	0.0	1.0
Lignoceric	1.9	0.2	2.8	0.3	0.2	0.4	1.4	0.1	2.1	0.0	0.0	0.0
Nervonic	0.0	0.0	0.0	11.6	15.6	9.6	3.9	4.6	3.5	0.0	0.0	0.0
DHA	0.0	0.0	0.0	54.3	66.4	48.3	18.6	19.9	17.9	15.5	25.1	10.7
ARA:DHA (*w*/*w*)							1.9	1.6	2.1	1.8	0.9	2.8

**Table 2:** Fatty acid composition (wt%) and positional distribution of structured TAGs from acidolysis at 45 °C and 1:3 ARA-oil:DHA-FFAs substrate molar ratio. The minor fatty acids contributing to the total fatty acid composition were capric acid, lauric acid and myristoleic acid.

Fatty Acid (wt%)	RM Lipase—8 h			CA Lipase—4 h		
	Total	*sn*-2	*sn*-1,3	Total	*sn*-2	*sn*-1,3
Myristic	0.6	0.3	0.7	0.5	0.4	0.6
Palmitic	13.0	4.2	17.4	11.1	4.2	14.5
Palmitoleic	0.5	0.4	0.5	0.4	0.4	0.4
Margaric	0.5	0.2	0.5	0.4	0.2	0.6
Stearic	6.3	2.1	8.4	7.1	1.7	9.8
Oleic	8.7	16.1	5.1	7.4	11.7	5.3
Linoleic	4.9	10.1	2.3	4.4	10.7	1.2
γ-Linolenic	3.0	4.3	2.3	2.8	3.7	2.3
α-Linolenic	0.8	0.9	0.8	0.9	0.9	0.9
Eicosenoic	0.3	0.6	0.2	0.4	0.4	0.3
Behenic	3.1	4.1	2.5	3.0	4.1	2.4
ARA	33.9	41.7	29.9	35.6	39.1	33.9
Erucic	1.1	0.4	1.5	1.3	0.4	1.8
Eicosapentaenoic	0.5	0.2	0.6	0.4	0.3	0.5
Lignoceric	1.1	0.4	1.5	1.3	0.4	1.8
Nervonic	3.8	2.4	4.4	4.1	3.9	4.2
DHA	17.3	11.0	20.5	18.7	16.9	19.6
ARA:DHA (*w*/*w*)	2.0	3.8	1.5	1.9	2.3	1.7

**Table 3:** Fatty acid composition (wt%) and positional distribution of TAGs from scale-up processes at 45 °C, 1:3 ARA-oil:DHA-FFAs substrate molar ratio, CA lipase, and 4 h reaction time. The minor fatty acids contributing to the total fatty acid composition were capric acid, lauric acid, and myristoleic acid.

Fatty Acid (wt%)	Scale-Up Processes		
	Total	*sn*-2	*sn*-1,3
Myristic	0.5 ± 0.0	0.4 ± 0.1	0.6 ± 0.0
Palmitic	9.3 ± 2.0	4.6 ± 0.2	13.4 ± 0.1
Palmitoleic	0.4 ± 0.0	0.4 ± 0.0	0.4 ± 0.0
Margaric	0.4 ± 0.0	0.1 ± 0.0	0.5 ± 0.0
Stearic	7.7 ± 0.6	2.0 ± 0.2	9.6 ± 0.4
Oleic	7.5 ± 0.0	11.7 ± 0.0	5.5 ± 0.1
Linoleic	4.6 ± 0.2	10.6 ± 0.1	1.3 ± 0.1
γ-Linolenic	2.7 ± 0.1	3.7 ± 0.0	2.2 ± 0.1
α-Linolenic	0.8 ± 0.0	0.9 ± 0.0	0.8 ± 0.0
Eicosenoic	0.3 ± 0.0	0.4 ± 0.0	0.3 ± 0.0
Behenic	3.4 ± 0.2	4.1 ± 0.0	2.7 ± 0.0
ARA	38.8 ± 2.4	39.3 ± 0.6	34.4 ± 1.1
Erucic	0.9 ± 0.0	0.6 ± 0.0	1.1 ± 0.0
Eicosapentaenoic	0.4 ± 0.0	0.2 ± 0.0	0.5 ± 0.0
Lignoceric	1.3 ± 0.0	0.3 ± 0.0	1.8 ± 0.0
Nervonic	3.5 ± 0.4	3.6 ± 0.2	4.1 ± 0.3
DHA	16.4 ± 2.3	16.5 ± 0.7	20.0 ± 1.4
ARA:DHA (*w*/*w*)	2.3	2.4	1.7

## Data Availability

The original contributions presented in the study are included in the article, further inquiries can be directed to the corresponding author.
